# Gabapentin-Induced Sub-Chronic Neurotoxicity in Rats and the Protective Role of Alpha-Tocopherol

**DOI:** 10.5146/tjpath.2025.14236

**Published:** 2025-09-30

**Authors:** Mohammad Abd-El-Same’e El-Kattan, Eman Saeed, Mahmoud Ahmed Khattab, Fatma Abdel Wahab Abdel Maksoud, Maha Emad Eldein, Nada Elsayed Abdel-Roaf, Walaa Awad, Ahmed Elshatory

**Affiliations:** Department of Forensic Medicine and Clinical Toxicology, Faculty of Medicine, Mansoura University, Mansoura, Egypt; Kuwait Poison Control Center, Ministry of health, Kuwait City, Kuwait; Department of Medical Histology and Cell Biology, Faculty of Medicine, Mansoura University, Mansoura, Egypt; Department of Medical Histology and Cell Biology, Faculty of Medicine, Mansoura National University, Gamasa, Egypt; Department of medical pharmacology, Faculty of Medicine, Cairo University, Cairo, Egypt; Department of Clinical and Chemical Pathology, Faculty of Medicine, Cairo University, Cairo, Egypt; Department of Pathology, Faculty of Medicine, Cairo University, Cairo, Egypt; Department of Forensic Medicine and Clinical Toxicology, Faculty of Medicine, Cairo University, Cairo, Egypt; Clinical Pharmacy Department, Abo El-Reesh Al Mounira Hospital, Cairo University, Cairo, Egypt; Kuwait Poison Control Center, Ministry of health, Kuwait City, Kuwait; Department of Forensic Medicine and Clinical Toxicology, Faculty of Medicine, Cairo University, Cairo, Egypt

**Keywords:** Gabapentin neurotoxicity, Misuse, Neurotoxicity, Alpha-tocopherol, Rats

## Abstract

***Objective: *** The past ten years have seen an increase in gabapentin (GBP) overuse and abuse in Egypt after pregabalin scheduling. Numerous studies have demonstrated the detrimental effects of pregabalin; nonetheless, GBP’s effects are minimal. The objective of this investigation is to study GBP-induced neurotoxicity in rats and the protective benefits of alpha-tocopherol (vitamin E “Vit E”).

***Material and Methods:*** Forty (40) adult male albino rats were randomly split into four groups: (10 rats each): Group I, which was subdivided into group Ia (5 rats), received a regular diet as a negative control; group Ib (5 rats) received corn oil as a positive control; group II received alpha-tocopherol; group III (GBP misuse); and group IV received GBP + alpha-tocopherol. The corresponding medicines were administered to every rat for fifty days. Neurobehavioral tests were performed on the day of scarification. Hippocampal tissues were collected for immunohistochemical and histological analysis.

***Results: *** Weight gain rose considerably by the end of the research in the drug-treated groups. In neurobehavioral tests, controls performed better and had higher locomotor indices. The group that misused GBP showed more deteriorated cells and more negative effects on hippocampal tissues. These histological alterations dramatically decreased with alpha-tocopherol therapy.

***Conclusion:*** GBP in high doses had neurotoxic effects, disrupted hippocampal tissues, and increased the number of degenerated cells. Alpha-tocopherol treatment significantly attenuated the deleterious effects induced by GBP.

## INTRODUCTION

The U.S. Food and Drug Administration (FDA) initially authorized GBP in 1993 for the treatment of epilepsy. GBP is one of the first-line drugs used to treat neuropathic pain (like multiple sclerosis, restless leg syndrome, and nerve pain that follows shingles). In 2002, the FDA approved GBP for use in treating post-herpetic neuralgia pain (1). GBP is one of the top ten most prescribed drugs in the US, despite only having two FDA-approved indications at this time. GBP has been administered since its inception for several off-label procedures and non-FDA-approved indications, including the treatment of agitation in dementia, drug and alcohol withdrawal, bipolar illness, diabetic nerve damage, anxiety, and fibromyalgia, migraine, and the list keeps getting longer (2). Actually, 95% of patients who were prescribed GBP were using it for off-label conditions, according to the research that looked at the managed Medicaid population (3).

Gabapentin is an amino acid that shares structural similarities with the neurotransmitter gamma-amino-butyric acid (GABA) (2). However, it does not directly interact with GABA receptors. GBP binds to and blocks the alpha-2-delta-1 subunit of presynaptic, voltage-gated calcium channels, which reduces the release of excitatory neurotransmitters. GABA has a calming impact on the brain by instructing neurons to slow down or cease firing. The GABA neurotransmitter and its associated drugs can have a significant effect on the brain because about 40% of the neurons respond to it. Gabapentin seems to function by modifying brain electrical activity that transmits pain signals between nerve cells, helping individuals with neuropathic diseases (4).

A wide range of adverse effects are known to occur with GBP as a result of intentional self-harm or suicidal thoughts, such as somnolence, exhaustion, dizziness, ataxia, involuntary muscle coordination, vision distortion, asthenia, or physical weakness. While supratherapeutic doses of GBP can lead to sedation, dissociation, numbness, contentment, relaxation, unrestrained conduct, empathy, and audio/visual hallucinations. Furthermore, gabapentin is known to cause abusers to become more gregarious, talkative, and unrestrained. The use of gabapentin may cause dissociative symptoms (like those observed with dextromethorphan) that contribute to its potential for abuse (5).

Concern over gabapentin usage and overuse is growing. Numerous cases of GBP abuse in combination with opiates, alcohol, cocaine, and benzodiazepines to enhance their effects and as a substitute therapy to lessen the effects of withdrawal when the abused substances are not accessible, according to several studies in the literature (6). Moreover, people with a history of opioid use disorder (OUD) are 22% more likely to take gabapentin in addition to opioids than those without OUD (7).

The US Drug Enforcement Administration (DEA) classifies pregabalin as a Schedule V medication, which is characterized as having a lower risk for abuse than Schedule IV medicines. However, gabapentin is not currently a federally banned narcotic. Nonetheless, gabapentin is scheduled in several states, including Virginia, West Virginia, Kentucky, Tennessee, Michigan, and North Dakota (8). Furthermore, several states have mandated gabapentin monitoring as part of their statewide prescription drug monitoring programs (PDMP), including Minnesota, Massachusetts, and Wyoming (9).

In previous studies, the potential abuse of gabapentin has been a contentious issue. Gabapentin has been classified as relatively safe despite the danger of abuse, even at dosages as high as 50,000 mg and up to 90,000 mg (10), which are both far higher than the FDA-recommended maximum dose of 3600 mg/day (11). Nonetheless, it has been suggested that the euphoric and mood-altering effects of the 3600 mg/day dose are what motivate the need for more frequent dosing (12).

It is well known that alpha-tocopherol (Vit E) is a strong antioxidant that regulates the expression of genes related to oxidative damage, inflammation, cell division, and apoptosis (13,14). Concurrent use of an antioxidant agent can stop the cytotoxic xenobiotic’s free radicals from damaging cells (15). Previous studies have examined the potential ameliorative effect of Vit E in connection to the hepatotoxicity of cyclophosphamide (16), the hepatic veno-occlusive effect of chemotherapy (17), the nephrotoxicity of gentamicin (18), and the renal impairment associated with cisplatin (19).

In Egypt, gabapentinoid drug misuse increased in the last decade. Although many studies have confirmed the neurotoxic effects of pregabalin, those of GBP are minimal. As far as we are aware, no previous study has looked at how Vit E mitigates the negative effects of chronic GBP use on the brain. The aim of the current investigation is to understand the increase in GBP prescription and potential for abuse, recognize the risks involved in its prescriptions with opioids, and clarify Vit E potential preventative efficacy against long-term GBP-induced neurotoxicity.

## MATERIAL and METHODS

### Study Locality

This prospective randomized controlled experimental investigation was carried out at the Research Institute of Ophthalmology’s Animal House in compliance with the Cairo University Faculty of Medicine’s Animal Care and Use Committee (CU-IACUC). Cairo University in Egypt granted approval to (CU-IACUC) (code number: CU-IΙΙ-F-78-22). The National Research Council’s Guide was adhered to in the handling and utilization of laboratory animals in this study.

### Animals

The present study involved forty (40) male albino rats in good health, weighing between 200 and 250 grams. Under typical laboratory conditions, which included a 12-hour cycle of darkness and light and a constant temperature of 25°C, they were housed in plastic cages. Water and the typical laboratory diet (libitum and pellets) were utilized for feeding. Four groups were randomly selected from among the animals.

### Instruments

#### ANY-Box® (USA: Stoelting Company)

The ANY-box is a behavior device with multiple configurations. It was used to assess the neurobehavioral changes. ANY-box is composed of two components: a base and a core. The ANY-box base comes with a camera so that the animals can be monitored. Mice were put through a variety of tests in different cages (20).

#### Drugs and Chemicals

■ Gabapentin (Neurontin® powder, 300 mg cap, Pfizer, Cairo, Egypt, under license from Pfizer Inc., USA).

■ Vitamin E (Vitamin E cap 1000 mg from Pharco Pharmaceuticals Cairo, Egypt).

■ Corn oil (Pharco Pharmaceuticals Cairo, Egypt).

### Study Design

#### 1. Group Ӏ: controls (10 rats)

■ **Group Ӏa negative control (5 rats):** each rat received a normal diet (libitum, pellet and water) orally by gavage method for 50 days.

■ Group** Ӏb positive control (5 rats): **each rat received one ml of corn oil orally by the gavage method for 50 days.

#### 2. Group II (Vit E): (10 rats)

Each rat was gavaged with Vit E dissolved in corn oil at a dose of 100 mg/kg/day or 20 mg/day for 50 days (21–23).

#### 3. Group III (GBP misuse): (10 rats)

For three days, each rat received an oral gavage of (6.48 mg/day) or (32.4 mg/kg/day) of GBP dissolved in distilled water. Every three days, the dose was gradually increased until the end of the 30-day period, when the dependent dose (64.8 mg/day) or (324 mg/kg/day) was reached. Furthermore, for the next 20 days, this last dependent dose was given every day (4,24,25).

#### 4. Group IV (GBP + Vit E): (10 rats)

For three days, each rat was given GBP in distilled water (6.48 mg/day) or (32.4 mg/kg/day) plus Vit E in corn oil (100 mg/kg/day) or (20 mg/day) orally via gavage. After three days, each rat continued to receive the same dosage of Vit E every day while the dose of GBP was escalated by adding the initial dose every three days until reaching the dependent dose (64.8 mg/day) or (324 mg/kg/day) when the 30-day period was over. Additionally, the last dependent dose of GBP with the same dosage of Vit E was continuously administered daily for another 20 days (23,25).

Using the Paget equation, the starting dosage of GBP was equivalent to the daily therapeutic dose of 360 mg/day. The dependent dose is equal to the dose that produces dissociative effects and the desirable euphoria in human addicts, which is 3600 mg/day. The equivalent dosage for a 200 g rat is 18/1000 times the usual therapeutic daily dose for a human adult (25–27). While alpha-tocopherol was administered at a concentration that is equivalent to the tolerable upper intake levels in adult humans according to the previous equation (21,22,26).

### Measurements

At the end of the experiment, the weight of the rats in each of the study groups was measured and compared with the weight at the beginning of the study (28).

### Sampling

The animals were euthanized by cervical decapitation to avoid any chemical injury or damage to the brain (29,30). At the end of the experiment, the brains were carefully retrieved and cleaned in normal saline. The hippocampal tissues were taken and then fixed in 10% neutral buffered formalin and were processed to prepare 5-µm-thick paraffin sections for both histological light microscopic and immunohistochemical studies.

#### 1. Animal Neurobehavioral Tests

At the conclusion of the study, the locomotor activity of the rats was evaluated by the use of ANY-box tests, e.g., open field test (20). Numerous standard behavioral tests, including the open field test, the dark and light test, and the novel object identification test, were automated and carried out. A video tracking system designed to automate testing in behavioral investigations was used to assess neurobehavioral tests (31).

#### 2. Histological and Immunohistochemical Studies

a) **Light microscopic study: **5um paraffin slices were acquired and stained using the following methods:

■ **Haematoxylin and Eosin (HX&E) stains:** to illustrate the overall histological structure (32).

▶ **Histopathological Scoring: **The score for hippocampal histopathology was identified in ten randomly selected non-overlapping fields. The score included some histological alterations in the hippocampus proprius and dentate gyrus (DG), degeneration of the pyramidal cell layer and bleeding and/or infiltration within the molecular layer. The score ratings were as follows: high damage (+++), moderate damage (++), mild damage (+), and no lesion (-) (33) (Table 1).

**Table 1:** Comparison of histopathological changes among the studied groups

	**Groups**			
**Parameters**	**Group I** **Control** **(n=10)**	**Group II** **Vit E** **(n=10)**	**Group III** **GBP misuse** **(n=10)**	**Group IV** **(GBP + Vit E)** **(n=10)**
Shrunken, pyknotic neurons	-	-	++	+
Hippocampus disorder	-	-	++	-
Ghost cell	-	-	++	+
Vacuolation	-	-	+	-

**n:** Number of rats; **GBP:** Gabapentin; **Vit E: **Vitamin E.

■ **Congo red stain:** to examine the β-pleated-sheet structure of amyloid (a histochemical marker of immune degeneration) (34).

■ **Periodic acid Schiff stain (PAS stain):** to evaluate glycogenosis and other metabolic disorders linked to neurodegeneration (35).

b) **Immunohistochemical Study: **Paraffin sections were immunologically stained for**:**

■ P-53 antibodies (marker for apoptosis and DNA integrity) (rabbit polyclonal anti-rat antibody against p53 (Abcam, ab131442; Cambridge, MA, USA) (36).

■ **Morphometric analysis: **The quantity of P-53 positive cells will be examined using ImageJ 1.47v software (National Institutes of Health, USA). Each rat will have five perceptive non-overlapping fields from immunostaining sections evaluated during the analysis process. The mean of the five readings will be used to represent each rat.

### Data Management and Statistical Analysis

The data collected were coded, processed, and analyzed with Statistical Package for the Social Sciences (SPSS) version 27 for Windows® (SPSS Inc., Chicago, IL, USA). The normality of distribution for the analyzed variables was tested using the Kolmogorov-Smirnov test, assuming normality at P > 0.05. The collected data were summarized in terms of median and interquartile range (IQR) as appropriate for nonparametric data and as number and percentage for qualitative data. The Friedman test was used to compare non-parametric quantitative data for repeated measures, followed by post hoc multiple comparisons using Bonferroni adjusted tests to detect the significant pairs. The one-way analysis of the variance (one-way ANOVA) was used to compare normally distributed quantitative variables. The Kruskal-Wallis test was used as a test of significance to compare independent non-parametric quantitative data. Significant Kruskal-Wallis was followed by an adjusted Bonferroni test to detect significant pairs. All tests were two-sided. The accepted level of significance in this work was p < 0.05; p ≤ 0.001 was considered highly statistically significant (HS), and p > 0.05 was considered non-statistically significant (NS).

## RESULTS

### 1) Effect of Long-Term Gabapentin Administration on Rat Weight

The four groups’ initial body weights were statistically similar. Table 2 displays each group’s starting and ending body weights along with their weight gain percentage. At the end of the trial, the GBP misuse group (G III) and Group IV (GBP + Vit E) had significantly higher body weights (p < 0.001). Groups (G III, IV) had a considerably higher body weight change percentage than other groups (G I, II) (Table 2).

**Table 2:** Comparison of body weight among the studied groups

** **	**Groups**				
**Parameters**	**Group I** **Control** **(n=10)**	**Group II** **Vit E** **(n=10)**	**Group III** **GBP misuse** **(n=10)**	**Group IV** **GBP + Vit E** **(n=10)**	**Significance test**
Initial body weight (g) Mean ± SD	195.50 ± 9.26	194 ± 10.22	193 ± 10.59	196 ± 9.07	F= 0.197 P= 0.898
Final body weight (g) Mean ± SD	252.80 ± 8.34	251.50 ± 8.18	268 ± 11.35	265 ± 9.72	F= 7.829 P < 0.001*
P1		0.990	0.005*	0.033*	
P2			0.002*	0.015*	
P3				0.894	
Percent of change (%) in body weight Mean ± SD	29.41 ± 3.16	29.77 ± 3.02	38.96 ± 2.55	35.27 ± 2.25	F= 27.605 P < 0.001*
P1		0.991	< 0.001*	< 0.001*	
P2			< 0.001*	< 0.001*	
P3				0.025*	

**n: **Number of rats; **F:** One-Way ANOVA; **P:** Tukey-HSD post hoc test; *: Statistically significant (p ≤ 0.05); **P1:** Significance in relation to G I; **P2:** Significance in relation to G II; **P3:** Significance in relation to G III; **GBP:** Gabapentin; **Vit E:** Vitamin E.

### 2) Effect of Long-Term Gabapentin Administration on Brain Weight

There were no discernible changes in brain weight in the GBP-treated groups when compared to the other groups (G I, II) (Table 3).

**Table 3:** Comparison of brain weight and relative brain weight among the studied groups

** **	**Groups**				
**Parameters**	**Group I** **Control** **(n=10)**	**Group II** **Vit E** **(n=10)**	**Group III** **GBP misuse** **(n=10)**	**Group IV** **GBP + Vit E** **(n=10)**	**Significance test**
Brain weight (g) Median (Range)	1.5 (1.5 – 4.5)	1.5 (1 – 4)	1.6 (1 – 3)	1.4 (1 – 3)	KW = 8.442 P = 0.038*
P1		0.962	0.032*	0.112	
P2			0.038*	0.642	
P3				0.146	
Relative brain weight (g) Median (Range)	0.006 (0.006–0.018)	0.006 (0.004–0.017)	0.005 (0.004–0.011)	0.005 (0.004–0.011)	KW=9.392 P=0.025*
P1		0.960	0.020*	0.045*	
P2			0.029*	0.052	
P3				0.326	

**n:** Number of rats; **KW:** Kruskal Wallis test; *: Statistically significant (p ≤ 0.05); **P1: **Significance in relation to G I; **P2:** Significance in relation to G II; **P3:** Significance in relation to G III; **GBP: **gabapentin; **Vit E:** vitamin E.

### 3) Animal Neurobehavioral Tests

The neurobehavioral functions were assessed for each animal. The open field test aimed to evaluate the locomotor activity and to assess mainly anxiety and irritability as signs of neurotoxicity through the number of midzone crosses, time spent in the peripheral zone, and number of immobile episodes. Irritable mice avoided the central zone and preferred the peripheral zone with fewer immobile episodes. In the dark and light test, irritable mice spent more time in dark zones. In the new object recognition test, irritable mice spent less time exploring new objects (Table 4).

**Table 4:** Neurobehavioral test results among the studied groups

**Rats** **(n=30 )**	**Group I** **Control (n=10)**	**Group II** **Vit E (n=10)**	**Group III** **(GBP misuse) (n=10)**	**Group IV** **(GBP + Vit E) (n=10)**	**p** **value**
**Open field test** Number of midzone crosses Peripheral zone time “min” Number of immobile episode	109 ± 14.12 4.5 ± 0.07 11 (7–13)	112 ± 17.12 4.4 ± 0.08 12 (7–14)	70.60 ± 11.66 * 4.76 ± 0.13 * 7 (4–9) *	41.40 ± 5.5*+ 3 ± 0.1*+ 17 (12–17) *+	< 0.001 **P**
**Dark and light test** Time spent in dark-zone	9 (7–13)	8 (7–11)	15 (12–17) *	5 (4–9) *+	< 0.001** P**
**New object recognition test** Time spent exploring new objects	6.62 ± 0.58	7.92 ± 0.22	2.41 ± 0.33 *	4.50 ± 0.37 *+	< 0.001** P**

**n:** Number of rats; **GBP:** Gabapentin; **Vit E: **Vitamin E; *: Significance in relation to G I & G II; ***+*** **: **Significance in relation to G III; **P:** Statistically significant (p ≤ 0.05).

### 4) Evaluation of Histopathological and Immunohistochemical Changes

#### a) HX&E stain

**Group I (Control Group)**

**Figure 1:**
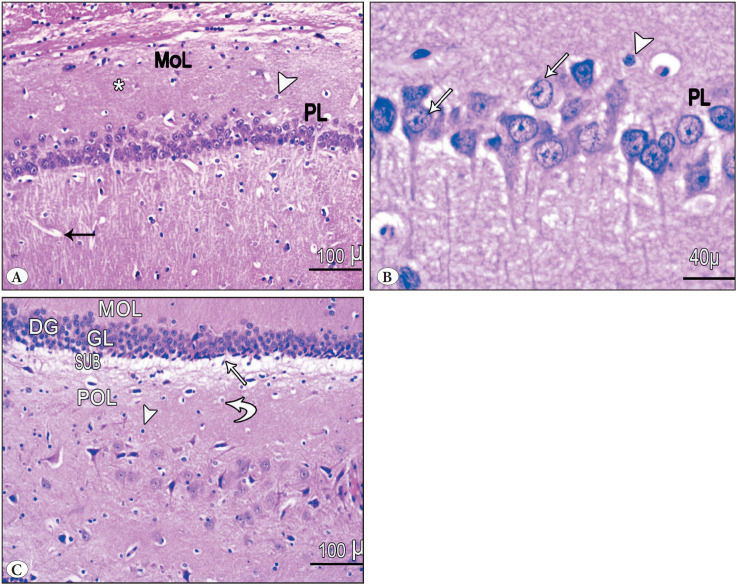
Photomicrographs of H&E-stained sections in the hippocampus of control group I. **A,B)** shows the hippocampus proprius. The hippocampus proprius was formed of pyramidal cell layer (PL) and molecular layer (MoL). Both appear to be normal in shape. The hippocampus reveals the normal structure of pyramidal cells (arrows), astrocytes (arrow head), and fibers (*). The pyramidal cell layer (PL) exhibits closely packed cell bodies of the pyramidal neurons (arrows) that are regularly arranged in 3 to 4 rows and appear small with vesicular nuclei, prominent nucleoli, and scanty cytoplasm. **C)** shows the dentate gyrus (DG). The DG consisted of molecular, granular, and polymorphic layers. The granular layer (GrL) contained compactly arranged layers of granule cells with vesicular nuclei and prominent nucleoli. Spindle-shaped immature cells (arrow) with oval darkly stained nuclei were seen in the subgranular zone. The polymorphic layer (PoL) contains both lightly (curved arrow) and darkly stained glial cells (arrow head).

The hippocampal region consisted of hippocampus proprius and dentate gyrus (DG). The hippocampus proprius is formed of the pyramidal cell layer (PL) and molecular layer (MoL). The molecular layer was formed of neuroglial cells and blood vessels. The pyramidal layer was formed of pyramidal cells that appeared as large, loosely packed triangular cells with vesicular nuclei and prominent nucleoli. Each cell showed an apical dendrite ramifying upward and basal dendrites (Figure 1). The DG consisted of molecular, granular, and polymorphic layers. The polymorphic layer showed scattered polymorphic nuclei. The granule cell layer (GRL) contained compactly arranged layers of granule cells with vesicular nuclei and prominent nucleoli. Spindle-shaped immature cells with darkly stained oval nuclei were seen in the sub granular zone (Figure 1).

**Group II (Vit E)**

**Figure 2:**
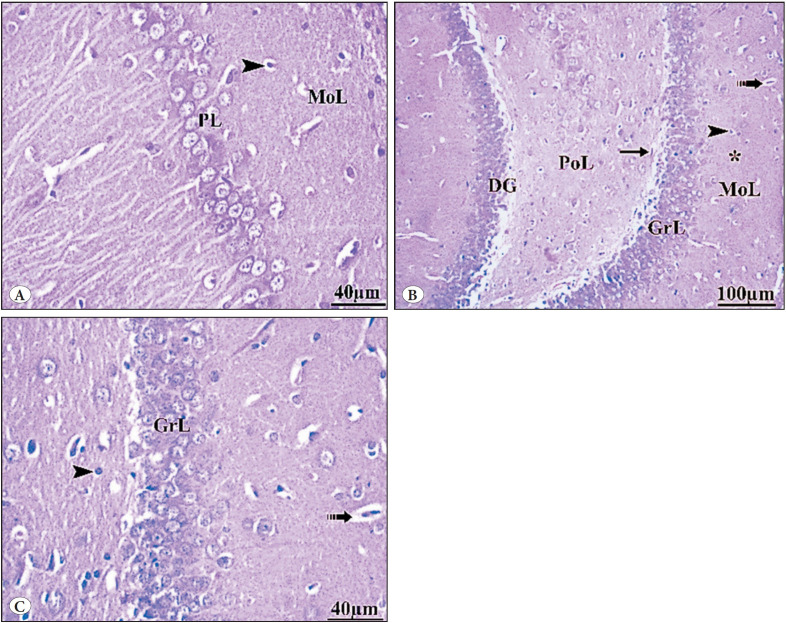
Photomicrographs of H&E-stained sections in the hippocampus of Vit E group II. **A,B)** The hippocampus’s pyramidal cell layer (PL) and molecular layer (MoL) are more or less similar to the control group I. Glial cells (head arrow) live alongside ordinary blood capillaries on a pink neuropil (*) background formed of neuronal and glial cell processes. **B,C)** show the dentate gyrus (DG). The granule cell layer (GrL) in the dentate gyrus (DG) is well-defined. The GrL displays the aggregation of granule cell bodies, which range from spherical to oval. Molecular layer (MoL), polymorphic layer (PoL), microglia (head arrow), and blood vessel (dotted arrow) are also seen with intact form. Immature neurons (arrow) in the subgranular layer were seen.

The hippocampus’s pyramidal cell layer (PL) and molecular layer (MoL) appeared more or less similar to control group I (Figure 2). The granule cell layer (GrL) in the dentate gyrus (DG) was well-defined. Molecular layer (MoL), granular layer, and polymorphic layer (PoL) were seen with intact form. Immature neurons (arrow) were seen in subgranular layer (Figure 2).

**Group III (GBP misuse)**

**Figure 3:**
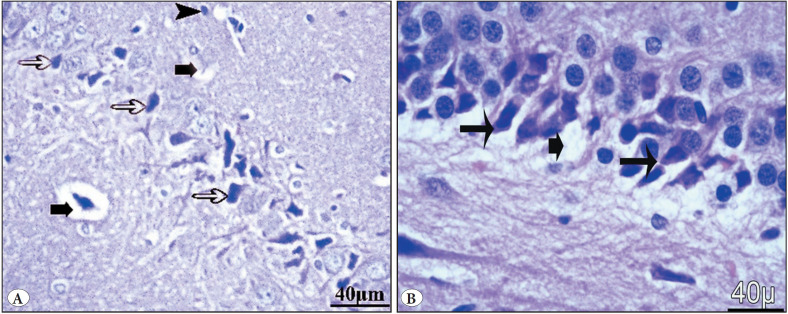
Photomicrographs of H&E-stained sections in the hippocampus of the GBP misuse group III. **A)** Shows the hippocampus proprius. The hippocampus proper appeared disorganized with degenerated pyramidal cells. The molecular layer appeared vacuolated with many neuroglia cells surrounded by empty spaces (arrow head). Loss of the normal arrangement of the pyramidal cells (PL) was evident. The cells were disorganized, dark, irregular in shape, and shrunken cells with darkly stained nuclei that appeared surrounded by large vacuolated pericellular spaces (hollow arrows) were also found. Many vacuoles are also observed (thick arrows). **B)** shows the dentate gyrus (DG). The granular cell layer (GrL) was disturbed with many dark-stained nuclei (black arrows) surrounded by empty spaces (thick arrows).

The hippocampus proper appeared disorganized with degenerated pyramidal cells. The molecular layer appeared vacuolated with many shrunken neuroglia cells surrounded by empty spaces. The hippocampus proprius showed numerous small shrunken pyramidal cells with darkly stained nuclei and cytoplasm. (Figure 3). The dentate gyrus showed many shrunken, darkly stained granule cells, in addition to normal granule cells with vesicular nuclei. The granular cell layer was disturbed with many dark-stained nuclei (Figure 3).

**Group IV (GBP + Vit E)**

**Figure 4:**
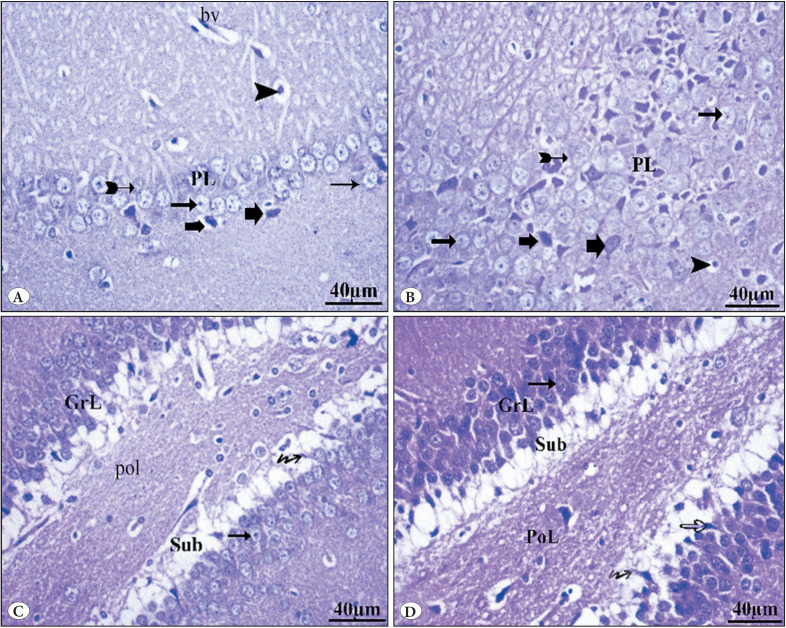
Photomicrographs of H&E-stained sections in the hippocampus of the GBP plus vit E group IV. **A,B)** show a normal molecular layer with neuroglial cells (arrowhead) lying along blood capillary (bv). Most of the pyramidal cells in the hippocampus appeared normal with vesicular nuclei (thin arrows). few degenerated cells with darkly stained nuclei that appeared surrounded by large vacuolated pericellular spaces (thick arrows). Degenerated neurons without nuclei (bifid arrows) were also seen. **C,D)** show the dentate gyrus (DG). Most granule cells in granular cell layers (GrL) appear normal (arrows), while others are degenerated cells (hollow arrow). Polymorphic layers (PoL) with intact neurons are still present. Immature neurons (wavy arrows) in the subgranular layer (Sub) are also seen. Notice normal granule cells (thin arrows).

Group IV showed relative improvement and a moderate degree of disorganization in between the pyramidal layer. The molecular layer appeared normal with neuroglial cells lying along. Most of the pyramidal cells in the hippocampus appeared normal with vesicular nuclei. A few degenerated cells with darkly stained nuclei surrounded by large vacuolated pericellular spaces were also seen. Degenerated neurons without nuclei were also seen (Figure 4). In the dentate gyrus (DG), most granule cells in granular cell layers appear normal, while others are degenerated cells. The polymorphic layers with intact neurons were still present. Immature neurons in the subgranular layer are also seen (Figure 4).

#### b) Congo red stain

**Figure 5:**
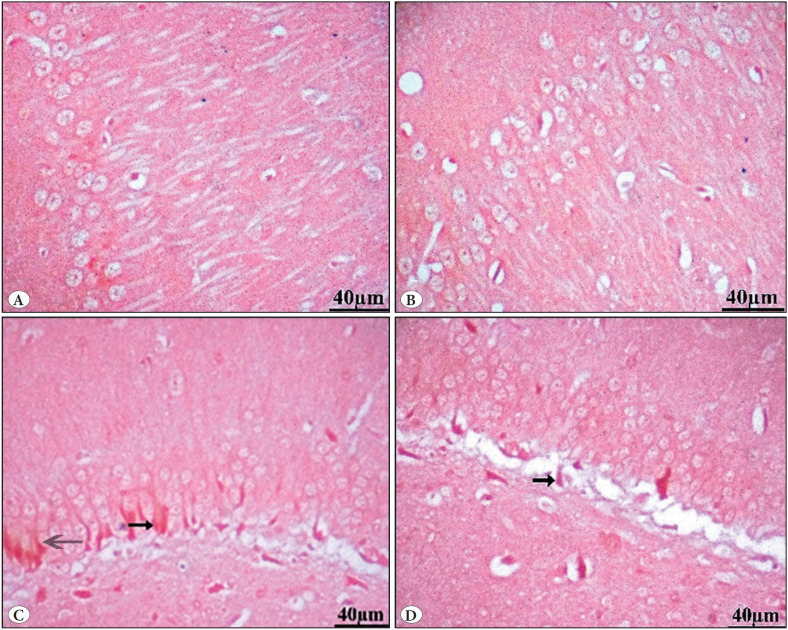
Photomicrographs of Congo red-stained sections in the hippocampus of **(A)** group I control showed negative Congo red stain in pyramidal neurons, **(B)** group II (vit E group) showed negative Congo red stain in pyramidal neurons, **(C)** group III GBP misuse showed many Congo red-positive pyramidal cells, and **(D)** group IV (GBP + Vit E) showed mild localized areas with few Congo redpositive pyramidal cells.

Group I (control) showed a negative Congo red stain in the pyramidal neurons. Group II (Vit E) showed a negative Congo red stain in pyramidal neurons. The GBP misuse group (Group III) showed many positive Congo red deposits. Group IV (GBP + Vit E) showed mild localized areas with few Congo red-positive pyramidal cells (Figure 5, Table 5).

**Table 5:** Comparison of PAS and cong red stains percent area among the studied groups

	**Groups**				
**Parameters**	**Group I** **Control (n=10)**	**Group III** **Vit E (n=10)**	**Group II** **GBP misuse (n=10)**	**Group IV** **GBP + Vit E (n=10)**	**Significance test**
Percent area of PAS stain	0.766 (0.350 – 0.970)	1.24 (0.729 – 2.77)	2.77 (1.88 – 4.65)	1.34 (1.115 – 1.959)	KW = 28.866 P < 0.001*
P1		0.025*	< 0.001*	0.019*	
P2			< 0.001*	0.910	
P3				< 0.001*	
Percent area of cong red stain	0.335 (0.181- 0.940)	1.879 (1.129 – 2.733)	3.08 (1.73 – 4.44)	1.844 (1.06 – 3.043)	KW = 27.664 P < 0.001*
P1		< 0.001*	< 0.001*	< 0.001*	
P2			< 0.001*	0.854	
P3				< 0.001*	

**n: **Number of rats; **KW: **Kruskal Wallis test; *: Statistically significant (p ≤ 0.05); **P1:** Significance in relation to G I; **P2: **Significance in relation to G II; **P3:** Significance in relation to G III; **GBP:** Gabapentin; **Vit E:** Vitamin E.

#### c) PAS stain

**Figure 6:**
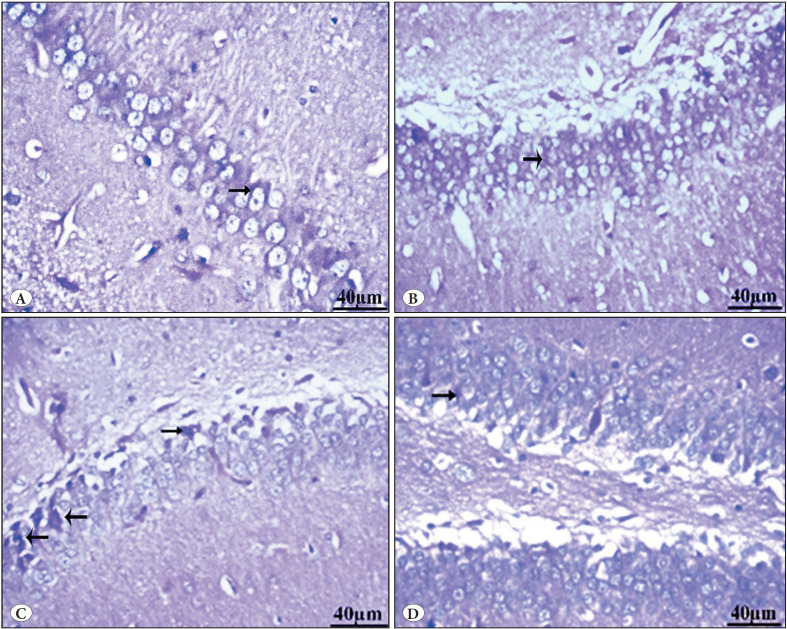
Photomicrographs of PAS-stained sections in the hippocampus of **(A)** group I (control) showed normal PAS reaction in the neurons (arrow), **(B)** group II (Vit E) showed normal PAS reaction in the neurons, **(C)** group III (GBP misuse) showed marked increase in PAS reaction in the neurons (arrows), **(D)** group IV (GBP + Vit E) showed mild PAS reaction in the neurons (arrows).

Group I (control) showed normal PAS reaction in the neurons. Group II (vit E) showed a normal PAS reaction in the neurons. Group III (GBP misuse) showed a marked increase in PAS reaction. Group IV (GBP + Vit E) showed a mild PAS reaction in the neurons (Figure 6, Table 5).

#### d) Immunohistochemical stains (P53)

**Figure 7:**
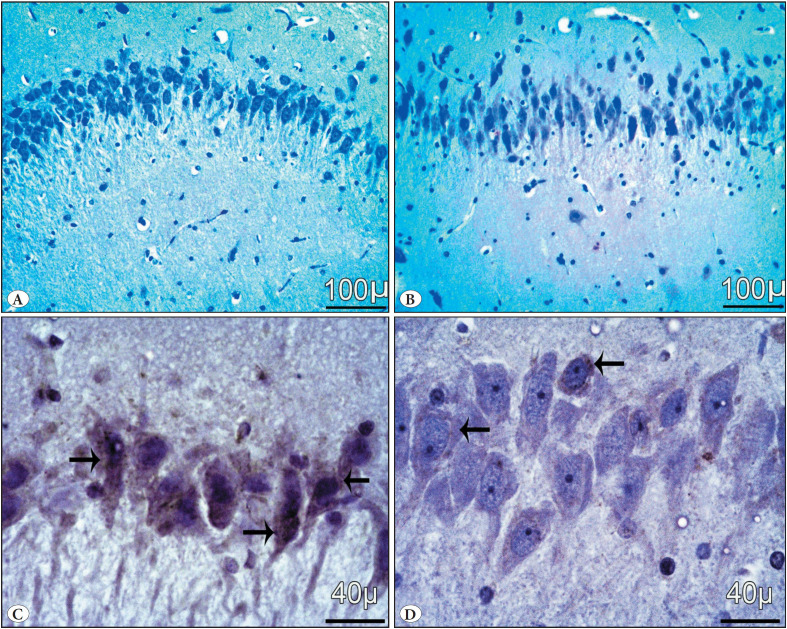
Photomicrographs of P53-stained sections in the hippocampus: **(A)** group I control and **(B)** group II Vit E showed undetectable reaction for the P53 gene. **(C)** Group III GBP misuse showed strong positive expressions of the P53 gene. **(D)** Group IV (GBP + Vit E) showed mild- moderate positive expressions of the P53 gene (arrows).

Group I (control) and group II (Vit E) showed negative immunoreactivity in neurocyte nuclei (Figure 7). The GBP misuse (Group III) showed brown immunoreactive staining of neurocyte nuclei, with marked expression of apoptotic cells (Figure 7). Group IV (GBP + Vit E) showed less positive immunoreactivity in some apoptotic cells (Figure 7).

## DISCUSSION

Addiction is considered a deadly and common illness that has several social and economic repercussions worldwide. The demand for gabapentinoid drugs, such as pregabalin and gabapentin, is high on the black market. Getting a prescription for GBP is not too difficult because of its extensive off-label use. The risk of GBP misuse increased in patients with a history of substance use disorder, especially those who are opioid dependent (5).

In this experiment, adult male albino rats were exposed to sub-chronic high doses of GBP to determine its neurotoxic potential. Alpha-tocopherol was also examined for its protective effect against GBP-induced neurotoxicity. We tried to replicate what happens with human addicts since they typically abuse GBP during the period of withdrawal from opioid medicines. Therefore, the authors are concerned about misuse of language in human addicts. The authors also intended to create an addiction model of GBP that included the dosage, duration, and manner of delivery. This aligns with many prior research studies (22,37,38) and case studies (39–41) that employ this addiction module.

At the end of the study, the abuse of high dosages of GBP was linked to notable weight gain. Comparing animals in groups III and IV to those in groups I and II, the former had a significant increase in body weight and final percentage of change. This could have happened since GBP can make rats feel hungrier and retain water.

According to DeToledo et al., 56% of patients receiving GBP for a year saw a gain in body weight (42). Based on its method of action, Tuluc et al. suggested that long-term GBP use may affect the voltage-gated calcium channels in the pancreatic β-cells that control insulin release (43). Buraniqi et al. reported in a recent systematic study that anticonvulsants, such as gabapentinoids, may change appetite and cause weight gain (44).

According to the present study, sub chronic misuse of high doses of GBP has caused a number of detrimental consequences on the hippocampal tissue, as evidenced by histological abnormalities when compared to control groups. Moreover, alpha-tocopherol has prevented the far greater histopathological damage that would have resulted from using GBP alone. This highlights the protective function of vitamin E.

Gabapentin-treated rats revealed structural alterations in their hippocampal tissues that were comparable to those seen in earlier research by Olaibi et al., which included a rise in neurodegenerative alterations such as the development of numerous disorganized, degraded pyramidal cells with irregular arrangements. Degenerated fragmented nuclei and many shrunken neuroglial cells gave the molecular layer a vacuolated appearance (45). This had been detected also by Badawy et al., who found that administration of GBP resulted in vacuolization and cavity formation in the brain tissue (25). Furthermore, there were obvious improvements in the histopathological appearance after the addition of vitamin E to GBP in group IV.

Looking for other histopathological stains, compared to the control group, the GBP misuse group displayed significantly more Congo red-positive deposits of β-pleated-sheet amyloid structure, indicating greater immunological degradation. Moreover, there has been a noticeable decrease in these amyloid deposits when vitamin E was added to GBP in group IV. In a similar vein, the GBP misuse group had numerous regions with positive PAS stain reactions, indicating increased glycogenosis and neurodegenerations that have improved in group IV.

This is the first study that we are aware of that reports oxidative stress, neurodegeneration, and amyloid deposits in the hippocampus during GBP misuse or abuse, as detected by both Congo red and PAS stains. We could not find any research that used Congo red or PAS stains to discuss the histological changes in the hippocampus.

Lastly, when comparing the immunohistochemical p53 stain results, the GBP misuse group (Group III) displayed more DNA disintegration and apoptosis because there were significantly more apoptotic cells stained positive for P53, whereas Group IV (GBP + Vit E) displayed fewer P53 +ve cells. Furthermore, comparable outcomes had been previously demonstrated (23,37,38).

The accumulation of p53 in the nucleus due to oxidative stress, genotoxic substances, and other circumstances causes it to attach to certain DNA sequences, activating the transcription of several genes linked to apoptosis. By activating caspase-3 and downregulating the anti-apoptotic BCL2, GBP caused apoptosis via the mitochondrial route (25,46,47). Furthermore, it damages DNA, raises ROS levels, and enhances the expression of the tumor suppressor p53, which in turn triggers the pro-apoptotic gene Bax and encourages apoptosis (23).

Other potential processes of GBP-induced neurotoxicity include the generation of free radicals like epoxide during the metabolism of GBP and the triggering of apoptosis, particularly in neural tube cells (48). Additionally, it was shown that GBP impairs the brain’s natural neuroprotective system, which is essential for neuronal survival during development (49).

The present study showed that combined treatment of Vit E and GBP revealed its neuroprotective effect, evidenced by overall improvements in the investigated hippocampal tissues with better arrangement of different pyramidal and molecular cell layers, improved ultrastructure, and decreased apoptosis, neurodegenerations, and amyloid and glycogen deposits.

As evidence of the ameliorating effect of Vit E, Mirzaei et al. found that Vit E treatment showed protective effects on oxidative stress and sperm apoptosis in diabetic mice (50). This was consistent with the study of Riffel et al., who concluded that Vit E modulates oxidative stress markers in the spinal cord of rats with neuropathic pain (51). Welson et al. found that Vit E protects against GBP-induced chronic hepatic and renal damage associated with the inhibition of apoptosis and tissue injury in rats (23). Liu et al. also found that Vit E could reduce the extent of mouse brain damage induced by combined exposure to formaldehyde (52). Vitamin E administration has also shown a neuroprotective effect on the central nervous system in streptozotocin-induced diabetic rats (53). Moreover, Vit E was found to protect the brain of rats from the deleterious effects of Huntington disease by improving energy metabolism (54). Vitamin E also has been reported to ameliorate the effect of Alzheimer’s disease (55).

The neuroprotective effect of Vit E may be attributed to different mechanisms. Alsemeh et al. showed that Vit E may rescue valproic acid-induced testicular injury in rats through induction of protective autophagy (56). Zhang et al. found that Vit E alleviates fungicide-induced toxicity in zebra fish by decreasing the oxidative stress level, restoring the functions of the heart and nervous system, and improving the immunity and mitochondria (57).

Yamadera et al. found that Vit E-coated dialyzer inhibits oxidative stress by decreasing malondialdehyde content, increasing the ratio of superoxide dismutase/malondialdehyde, enhancing the free radical scavenging activity, and decreasing the levels of pro-inﬂammatory cytokines (58).

Along with being a potent biological antioxidant, alpha-tocopherol also works in concern with other antioxidants to reduce free radicals. Furthermore, alpha-tocopherol functions similarly with several key components of the peroxidase-dependent antioxidant defense complex system, including the enzymes catalase and glutathione peroxidase (GPx) (14). Vit E halts oxidative damage through a variety of mechanisms. For instance, it scavenges free radicals, preventing lipid peroxidation and apoptosis (59).

## CONCLUSION

As far as we are aware, this is one of the few experimental studies that have investigated GBP’s potential for misuse/abuse and the protective role of Vit E in ameliorating GBP-induced neurotoxicity. The current work demonstrated that subchronic high doses of GBP caused detrimental histopathological and immunohistochemical changes in brain hippocampal tissues, suggesting that GBP may have addictive tendencies. Consequently, further rules must be put in place to limit the prescription of GBP. More research is required to further reassess the possibility of GBP being abused and misused, particularly in patients with a history of drug addiction.

## Funding

The research was self-funded by the authors.

## Conflict of Interest

The authors declare no conflict of interest
